# Detection of anti-SARS-CoV-2-Spike/RBD antibodies in vaccinated elderly from residential care facilities in Romania, April 2021

**DOI:** 10.3389/fepid.2022.944820

**Published:** 2022-09-20

**Authors:** Teodora Vremera, Florentina Ligia Furtunescu, Mihaela Leustean, Alexandru Rafila, Adina David, Iuliana Radu, Ana Maria Cornienco, Adina Gatea, Ciprian Ilie, Luminita Smaranda Iancu, Adriana Pistol

**Affiliations:** ^1^ECDC Fellowship Programme, EUPHEM Path, European Centre for Disease Prevention and Control (ECDC), Solna, Sweden; ^2^National Center for Surveillance and Control of Communicable Diseases, National Institute of Public Health, Bucharest, Romania; ^3^Department of Complementary Sciences, “Carol Davila” University of Medicine and Pharmacy, Bucharest, Romania; ^4^Seroepidemiological Diagnostic Laboratory, Regional Centre for Public Health Bucharest, National Institute of Public Health, Bucharest, Romania; ^5^“Grigore T. Popa” University of Medicine and Pharmacy, Iasi, Romania; ^6^Regional Centre for Public Health Iasi, National Institute of Public Health, Bucharest, Romania

**Keywords:** SARS-CoV-2 infection, COVID-19, anti-Spike/RBD IgG, antibody, elderly, residential care facility

## Abstract

**Introduction:**

SARS-CoV-2 infection rates and related mortality in elderly from residential care facilities are high. The aim of this study was to explore the immune status after COVID-19 vaccination in people 65 years and older.

**Methods:**

The study involved volunteer participants living in residential care facilities. The level of anti-Spike/RBD antibodies was measured at 2–12 weeks after complete vaccination, using chemiluminescent microparticle immunoassay (*SARS-CoV-2 IgG II Quant Abbott)*.

**Results:**

We have analyzed 635 serum samples collected from volunteers living in 21 Residential Care Facilities. With one exception, in which the vaccination was done with the Moderna vaccine, all volunteers received the Pfizer-Comirnaty vaccine. Individuals enrolled in the study had ages between 65–110 years (median 79 years). Of the people tested, 54.8% reported at least one comorbidity and 59.2% reported having had COVID-19 before vaccination. The presence of anti-S/RBD antibodies at a protective level was detected in 98.7% of those tested (*n* = 627 persons) with a wide variation of antibody levels, from 7.1 to 5,680 BAU/ml (median 1287 BAU/ml). Antibody levels appeared to be significantly correlated to previous infection (*r* = 0.302, *p* = 0.000).

**Conclusions:**

The study revealed the presence of anti-SARS CoV-2 antibodies in a significant percentage of those tested (98.7%). Of these, more than half had high antibody levels. Pre-vaccination COVID-19 was the only factor found to be associated with higher anti-S/RBD levels. The significant response in elderly people, even in those with comorbidities, supports the vaccination measure for this category, irrespective of associated disabilities or previous infection.

## Introduction

Severe Acute Respiratory Syndrome Coronavirus 2 (SARS-CoV-2) is responsible for the COVID-19 respiratory disease, the cause of the ongoing global pandemic with more than 263,000,000 confirmed cases and 5,200,000 deaths worldwide by December 1st 2021^1^. In Romania, as of December 1st 2021, there are more than 1,700,000 confirmed cases and 56,000 deathsdeaths (see text footnote 1).

Most cases of COVID-19 are mild to moderate and recover without specific treatment. Severe disease can occur in various proportion, with predilection in older people (aged 65 years and above), and in those with underlying medical conditions (1, 2). Severe evolution is caused by cytokine storm syndrome which is responsible for Acute Respiratory Distress Syndrome (ARDS), with an estimated mortality of 40%. Infected persons can rapidly develop an acute, diffuse and permanent inflammatory alveolar damage associated with hypoxemia (3).

SARS-CoV-2 infection rates and related mortality in elderly population in residential care facilities are high (4, 5). Residents of long term care facilities are at increased risk for negative outcomes of infection due to advanced age, immune system deficit, and the presence of underlying diseases (diabetes, cardiovascular disease, chronic respiratory disease, cerebrovascular disease, malignancy, and dementia) (6–8). The risk for transmission of infection and outbreaks in a closed institution is higher given the close contact between residents and care workers (5, 6). Numerous countries in Europe, and the United States of America reported high COVID-19 deaths in residential care facilities, with percentages between 19 and 72% of all SARS-CoV-2-related deaths (7).

Until vaccination became available, the best way to prevent infection was based on non-specific methods of prophylaxis that recommended hand hygiene, wearing a protective mask and keeping physical distance. In Romania, vaccination was high priority for the government. A national strategy has been approved by the government and national intersectoral committee was nominated to plan and implement it^2^. The National Coordinating Committee for Activities on Vaccination against COVID-19 constantly coordinates and monitors all activities carried out within the national vaccination campaign.

Vaccination campaign began on 27 December 2020 in 10 infectious disease hospitals, involved in the first line in the fight against COVID-19. According to the vaccination strategy, the first population group to receive the vaccine against COVID-19 was the medical staff from the public and private systems. Starting from January 15, Stage II of the vaccination campaign began, aimed at people over the age of 65, especially from residential facilities, people with chronic diseases regardless of age, as well as key personnel working in key areas (see text footnote 2).

Given that in elderly the immune response to vaccination could be weaker compared to younger individuals (8), we aimed in this study to explore the immune status after COVID-19 vaccination in people 65 years and older from Romania, in order to guide preventive measures. The objective was to determine anti-Spike SARS-CoV-2 IgG antibodies in serum samples collected from vaccinated elderly in residential care facilities, over a 6 months period.

## Materials and methods

We planned a real world study involving volunteer participants living in residential care facilities. We intended to measure the immune status at 2–12 weeks after receiving the second dose of vaccine. The immune status was assessed based on the level of anti-Spike/RBD antibodies.

### Sample size

Convenience sampling was used. Sample size was calculated at 540 using EPI Info, with 95% confidence level, a maximum loss to follow-up of 40% based on literature reports (9, 10) and taking into consideration the number of persons belonging to this population group that was scheduled for vaccination.

### Inclusion criteria

- People 65 years and older;
- People living in residential care facilities;
- People vaccinated with two doses of the Pfizer-Comirnaty or Moderna COVID-19 vaccine;
- Moment of specimen collection-−2 to 12 weeks after the second dose of vaccine.

### Exclusion criteria

People who did not sign the informed consent to be involved in the study.

### Serum specimen collection

Samples were collected during April 2021. Prior to serum collection, participants completed the questionnaire on the existence of associated pathologies that can influence the response to vaccination, such as immune suppressive conditions.

### Laboratory method

Detection of anti-Spike/RBD IgG was done using chemiluminescent microparticle immunoassay, according to manufacturer instructions (*SARS-CoV-2 IgG II Quant Abbott, ARCHITECT i System, USA)*, with 99.60% specificity and 99.35% sensitivity. In order to ensure the reproducibility, the testing of all samples was carried out at the National Institute of Public Health Laboratory, Bucharest. This assay allows the quantitative determination of IgG antibodies against the receptor-binding domain (RBD) of SARS-CoV-2 spike protein. Antibody level was measured in arbitrary units (AU/mL) and the results were expressed both in AU/mL and in binding antibody units (1 BAU/mL = 0.142 × AU/mL) using the manufacturers' conversion factors for the WHO International Standard^3^. (11). A positive result was considered at values >50 AU/mL (7 BAU/mL). Validation of the reagent kit by the manufacturer using plaque reduction neutralization titer (PRNT) allowed the identification of specific thresholds that have a 95% likelihood to correspond to stronger level of neutralizing activity^4^. Based on these thresholds, we divided our cases into five groups which we assume to have progressively higher immune response: group 1 (7-148 BAU/mL), group 2 (149-503 BAU/mL), group 3 (504-590 BAU/mL), group 4 (591-986 BAU/mL), group 5 (>987 BAU/mL). Thus, we analyzed the positive outcomes globally and by intervals, in relation to sex, age, comorbidities, BMI and previous SARS-CoV-2 infection.

### Data collection

Information on sex, age, associated pathologies, previous COVID-19 infection and previous antibody positive test before vaccination was collected *via* questionnaires that were filled in by the participants and validated by the formal caregivers, according to the patients' medical record.

Data on comorbidities included previous diagnosis of most common chronic diseases> cardio-vascular diseases (hypertension, congestive heart failure, coronary artery disease), cancer, diabetes mellitus, obesity, chronic renal failure, liver cirrhosis, autoimmune disease, dementia and depression Other pathologies could be mentioned in the questionnaire.

### Statistical analysis

Statistical analysis was done using SPSS software, with *p*-values < 0.05 considered statistically significant. Quantitative variables were analyzed as distribution, measures of central tendency (mean, median) and confidence interval. The normal distribution was analyzed using the Kolmogorov–Smirnov test. The medians were compared using Mann–Whitney U or Median test Categorical variables were analyzed using the Chi-square or Fisher test. We explored the association of the immune response with age, BMI, previous SARS-CoV-2 infection, length of the interval between vaccination and testing and the number of reported comorbidities, by using Pearson or Spearman linear correlations. We interpreted the association as significant if *p*-value was lower than 0.05.

### Ethical statement

The study was approved by the Ethics Commission of the National Institute of Public Health (No 3245/05.03.2021).

## Results

We have analyzed 635 serum samples collected from volunteers living in 21 Residential Care Facilities located in 7 out of 42 counties of the country (Alba, Braşov, Călăraşi, Cluj, Gorj, Mureş, and Suceava) from all main regions of the country (Central, South, North-East, and North-West).

With one exception, in which the vaccination was done with the Moderna vaccine, all volunteers included in the study received the Pfizer-Comirnaty vaccine.

### Characteristics of the study population

#### Age and sex distribution

Individuals enrolled in the study, among which 63.1% women, had ages between 65–110 years (median age 79 years), with a distribution significantly different from the normal one (*p* < 0.001, Kolmogorov Smirnov test). Women seemed to have more advanced age (median age 82 years vs. 74 in men, *p* < 0.001, Median test).

#### Associated reported pathologies

Of the people tested, 348 (54.8%) reported at least one comorbidity. Among the most frequently described were: cardio-vascular diseases (hypertension, congestive heart failure, coronary artery disease) (26.1%), obesity (18.7%), diabetes mellitus (14.3%) and dementia or depression (13.5%). Comorbidities were most prevalent in females (58 vs. 49% in females and males respectively, *p* = 0.028) (Table 1).

**Table 1:** Distribution of comorbidities by sex, Romania, April 2021.

**Comorbidity**	**Females**		**Males**		**Total**		*p*-value^*, **^
	**Number**	**% of total number of females**	**Number**	**% of total number of males**	**Number**	**% of total**	
Cardio-vascular diseases	108	26.9	58	24.8	166	26.1	0.553*
Obesity	83	20.7	36	15.4	119	18.7	0.098*
Diabetes mellitus	60	14.9	31	13.2	91	14.3	0.552*
Dementia/depression	64	15.9	22	9.4	86	13.5	0.019
Malignancy	12	3	17	7.3	29	4.6	0.012
Chronic renal insufficiency	4	1	7	3	11	1.7	0.127**
Autoimmune disease	4	1	0	0.0	4	0.6	NA
Liver cirrhosis	0	0.0	3	1.3	3	0.5	NA

^*^Chi-Square Test.

^**^Fisher's test.

#### Previous SARS-CoV-2 infection

Of the total number of participants, 376 (59.2%) reported having COVID-19 before vaccination. No significant difference was found among females and males (58.9% and 59.8% previously infected females and males respectively, *p* = 0.867).

Self-recorded nucleocapsid antibody testing before vaccination was available in 34 (5.4%) volunteers, that have performed the testing 3–6 months before the start of the study. All participants with positive nucleocapsid antibody test before vaccination (*N* = 33) also declared having had previous SARS-CoV-2 infection. Most (*N* = 18) were women.

#### Results of anti-spike/RBD IgG detection

The time from vaccination to antibody testing varied between 18 to 80 days, with a median of 74 days.

The presence of Spike antibodies at a protective level was detected in 98.7% of those tested (*n* = 627 persons). Of the participants with previous COVID-19 infection, one 92 years old female with autoimmune disease did not present antibodies at 2 months after vaccination.

The level of antibodies in the protected group showed a wide variation, from 7.1 to 5,680 BAU/ml, with a median of 1,287 BAU/ml. Considering the five groups resulting from the thresholds specified by the producer, more than half of our subjects showed a high level of protection (> 987 BAU/mL) belonging to the fifth group (Table 2).

**Table 2:** Distribution of antibody levels by sex, in the five groups, in Romania, April 2021.

**Sex**			**Unprotected**	**Protected**					**Total**
				**Group 1**	**Group 2**	**Group 3**	**Group 4**	**Group 5**	
	**Anti-S/RBD antibody level**	**AU/mL**	<50	50	1050	3550	4160	6950	
		**BAU/mL**	<7	7	149	504	591	987	
	**Correspondence with neutralizing antibody titer**		<20	20	80	160	250	640	
Males	No.		1	37	37	5	32	122	234
Females	No.		7	47	56	10	44	237	401
Total	No.		8	84	93	15	76	359	635
	%		1.3	13.2	14.6	2.4	12.0	56.5	100.0

Analysis by gender revealed that median level of antibodies was lower in males (1,085.2 vs. 1,404.1 BAU/mL in males and females respectively), but the difference did not reach the statistical significance (*p* = 0.090, median test). Also the distribution by gender among the five groups of study did not differ significantly (*p* = 0.35, Chi^2^ test).

Further on, we searched the association of the antibody response to some personal characteristics (age, BMI, previous SARS-CoV-2 infection, length of the interval between vaccination and testing and the number of comorbidities reported). Antibody levels appeared to be significantly correlated to previous infection (correlation coefficient *r* = 0.302, *p* = 0.000) and to the number of days from vaccination (*r* = −0.154, *p* = 0.000). There was a correlation with the number of comorbidities, but it did not reach significance level (*r* = −0.068, *p* = 0.088), possibly due to the small number of subjects reporting comorbidities. Age and body mass index appeared to be not-related to the antibody level (Table 3).

**Table 3:** Correlation^*^ of antibody levels to age, BMI, previous COVID-19, interval from vaccination and number of comorbidities, Romania, April 2021.

**BAU/mL**	**Age**	**BMI value**	**Previous SARS-CoV-2 infection**	**Days from vaccination**	**Number of comorbidities**
Correlation Coefficient	0.063	−0.031	0.302	−0.154	−0.068
*p*-values	0.111	0.444	<0.001**	<0.001**	0.088

^*^Spearman correlation.

^**^Statistical significance is met.

**Figure 1:**
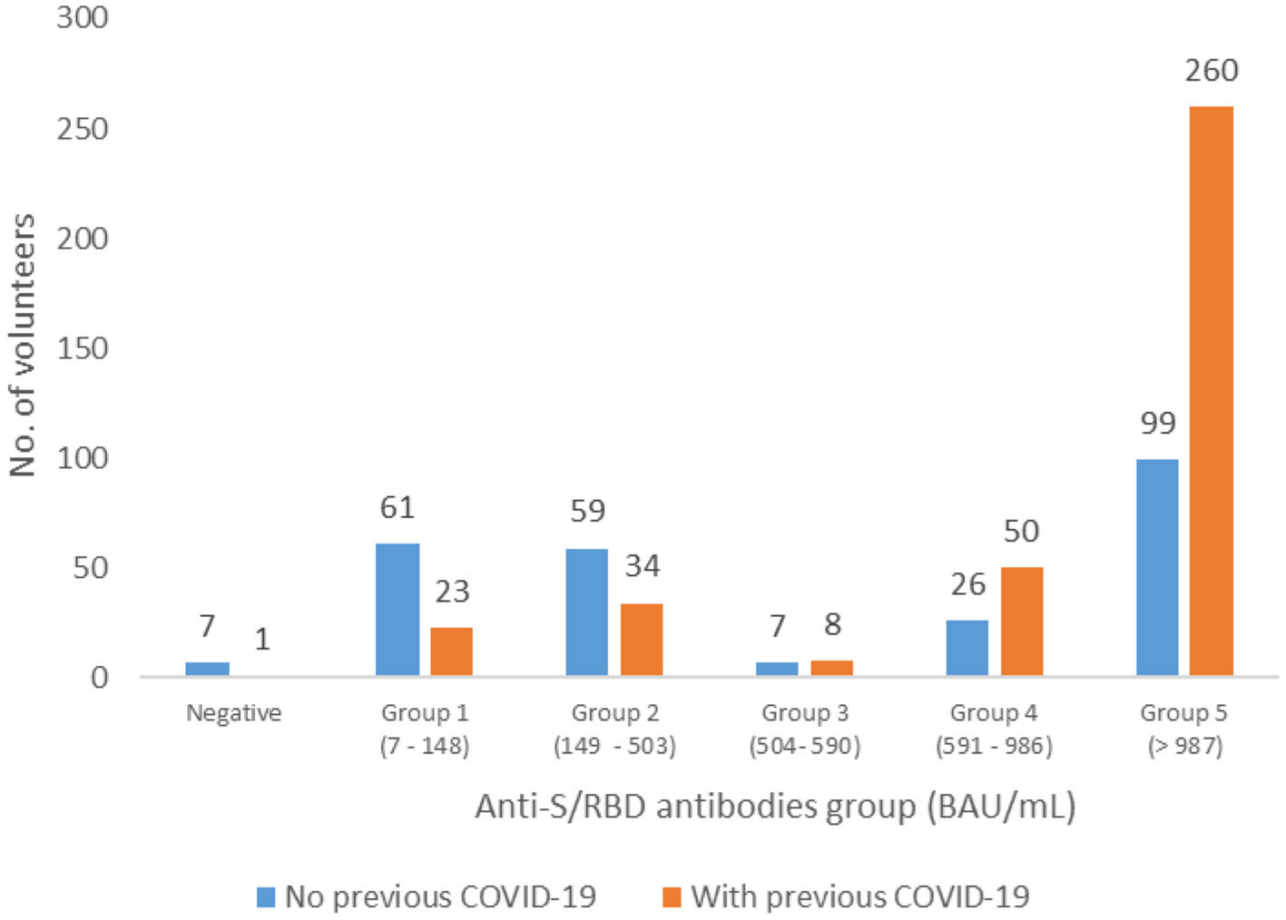
Distribution of the patients with and without previous COVID-19 infections in the five groups of anti-S/RBD antibody levels, Romania, April 2021.

Antibody values were significantly higher in those with previous COVID-19 [mean 2,362.5, 95% CI (2,170, 2,550), median 1,689.8] than in those without previous COVID-19 [mean 1,537.7, 95% CI (1,304.8, 1,770.6), median 523.7] (*p* < 0.00001, Mann Whitney *U*-test). Analysis of the five groups revealed that patients with previous COVID-19 had a predominance in those groups with higher immune response (groups 4 and 5), with a statistically significant difference among groups (p < 0.001, Chi^2^ test) (Figure 1). Spike antibody levels in those previously infected showed no significant difference between those with declared positive antibody test before vaccination compared to those without declared antibody test (*p* = 0.0718).

People with negative antibody test (*n* = 8) were predominantly women (*n* = 7), with ages ranging from 70 to 92 years old (median 83 years). All had at least one comorbidity such as cardio-vascular disease, diabetes mellitus, chronic renal failure, dementia or depression. The number of persons that were negative for anti-S antibodies was significantly higher in those that did not have previous COVID-19 infection (*p* = 0.006, Fisher test).

**Figure 2:**
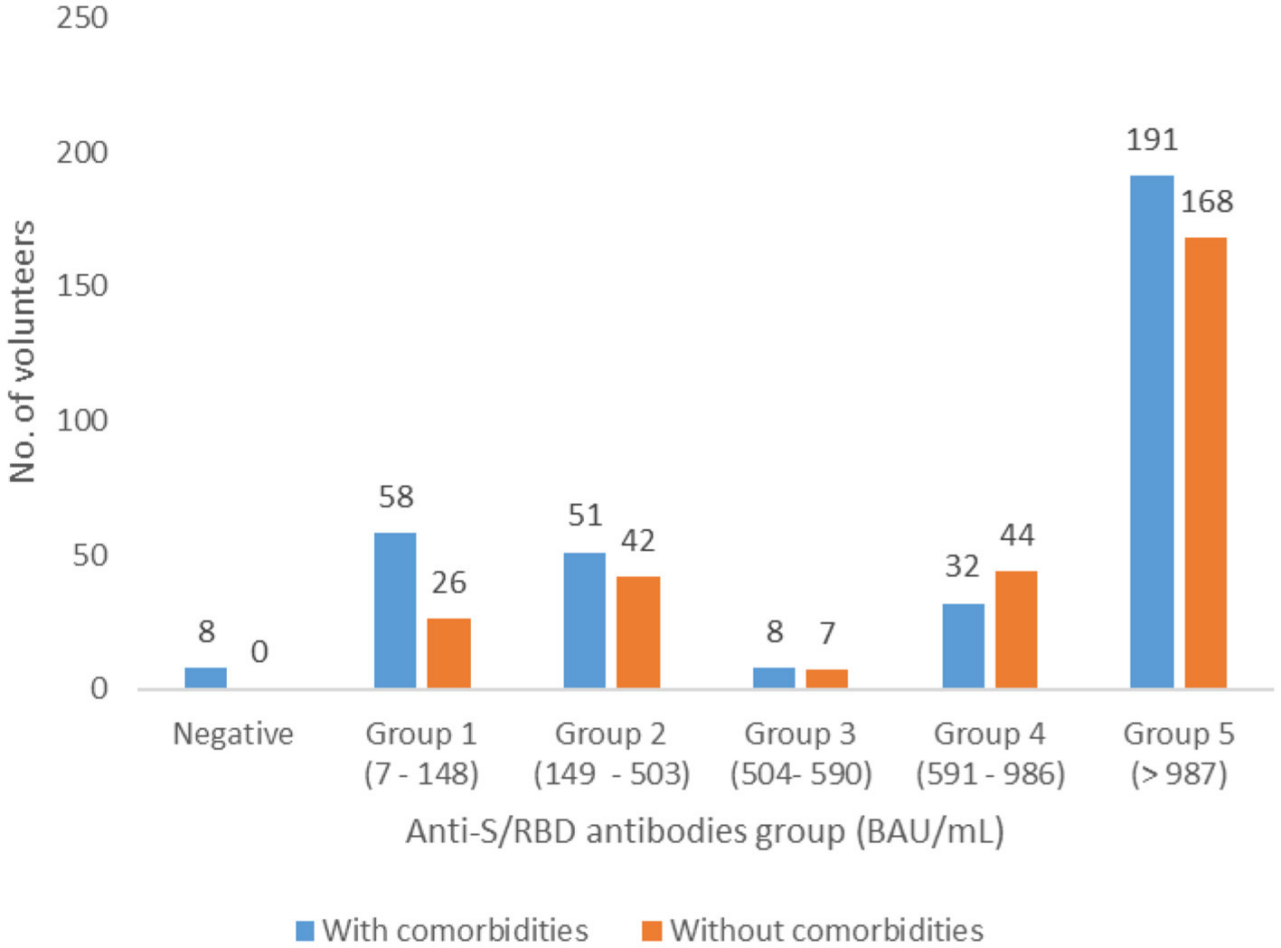
Distribution of the patients with and without associated pathologies in the five groups of anti-S/RBD antibody levels, Romania, April 2021.

Distribution of antibody levels for volunteers with associated pathologies did not show significant differences compared to those without comorbidities (*p* = 0.1422) (Figure 2).

## Discussions

Elderly individuals living in residential care facilities represent a particularly vulnerable population that has been disproportionately affected by COVID-19 and was one of the first groups targeted by vaccination (12). The immune response differs in each individual and is known to be influenced by age, sex, and associated pathologies (13). The decline of immune system functionality with age is linked with diminished response to vaccination and lower antibody levels (13).

Post-vaccine antibodies usually occur within 1 to 3 weeks after vaccination and only some of them are capable of also blocking the infection. Antibodies that block viral interaction with the receptor and are able to inhibit the virus are called neutralizing antibodies. Since the spike proteins on the surface of the virus allow the virus entry into the cell, neutralizing antibodies are predominantly produced against the spike proteins of SARS-CoV-2 (8).

A study by Lopez Bernal et al. in patients 70 years and older vaccinated with Pfizer-BioNTech reported a vaccine efficacy of 70% in 10–13 days after the first dose and 85–90% after two doses. Protection against symptomatic disease was also observed, with further protection against severe disease: 43% lower risk of emergency hospitalization and 51% lower risk of death after just one dose of vaccine. The observed protection was maintained >6 weeks (8, 14).

Our study showed the presence of anti-Spike antibodies in the majority of those tested (98.7%) with values above 987 BAU/mL, linked to high neutralizing antibody titer on cell cultures (>640)^3^, in 57.3% of positive samples. However, although anti-Spike antibodies correlate well with neutralizing activity, it is necessary to use plaque reduction neutralization test in order to demonstrate the neutralizing capacity and to quantify the titer of neutralizing antibodies. Also, anti-Spike antibodies appear both after infection and after vaccination. Since, in our study, the sample collection was done only after receiving both doses of vaccine and there was no evaluation of previous immune status, the antibody level detected can be influenced by a previous infection. This constitutes a limitation of the study that can be corrected by further testing serum samples for anti-nucleocapsid antibodies.

Hormonal differences and the presence of two X chromosome provide females with greater antibody response and greater inflammatory and antiviral responses compared to males (13, 15, 16). In our study, no significant differences were observed between females and males, probably explained by the drop in estrogen levels after menopause.

The presence of comorbidities can impact the humoral response after vaccination in the elderly population (17). Cardiovascular diseases (e.g., hypertension, congestive heart failure), end-stage renal disease, cancer, autoimmune diseases, metabolic disorders, chronic inflammatory processes, depression and dementia are relevant comorbidities that can cause dysfunctional immune system and aging serves as a significant aggravating factor (13, 17).

Our results showed no significant differences between those with comorbidities and those without comorbidities and this is in accordance with reports from literature that found that comorbidities, disability, sex, or cognitive impairment were not associated with different antibody levels after COVID-19 vaccination (12).

Increased antibody level was found in those with COVID-19 before vaccination and this is also in accordance with literature reports (12).

## Study limitations

Although information on vaccination and previous COVID-19 infection could be verified in the national online platform *Corona-forms*, information on associated pathologies and previous antibody test results were based on the correct completion of the questionnaires by the persons enrolled in the study and verified by the employed staff of the residential care facilities in accordance with the medical file of the patients.

Residents in long term care facilities represent a special population category and the results obtained cannot be extrapolated to the entire elderly population over 65 years. More than this, we used a convenience sample and the time from vaccination to antibody testing had a wide variation, with half of participants having 74–80 days from vaccination, which limits, again, the extrapolation of our findings.

Monitoring the persistence of antibodies and additional testing for cellular immunity and neutralizing activity are necessary in order to establish future conduct regarding prophylaxis measures in the elderly population in residential care facilities, which present certain particular features, both in terms of the increased risk of severe COVID-19 and in terms of the immune response. Anti-nucleocapsid antibodies need to be tested additionally to highlight the response to a possible infection.

In conclusion, the study revealed the presence of anti-SARS-CoV-2 antibodies in a significant percentage of those tested (98.7%). Of these, more than half (57.3 %) had high antibody levels (> 987 BAU/mL). Pre-vaccination COVID-19 was the only factor found to be associated with higher anti-Spike levels. The significant response in elderly people, even in those with comorbidities, supports the vaccination measure for this category, irrespective of associated disabilities and previous infection. Since immunogenicity after vaccination does not allow to draw conclusions on protection against infection or severe disease and since not all participants developed antibodies after vaccination, maintenance of non-pharmaceutical preventive measures in these settings is advised.

## Data availability statement

The raw data supporting the conclusions of this article will be made available by the authors, without undue reservation.

## Ethics statement

The study was approved by the Ethics Commission of the National Institute of Public Health (No 3245/05.03.2021). The patients/participants provided their written informed consent to participate in this study.

## Author contributions

TV designed the study, planned analyses, analyzed the data, and prepared the manuscript. ML supervised the laboratory testing. AR participated in designing the study. AD, IR, AC, AG, and CI carried out laboratory testing. FF participated in data analysis and preparing draft of the manuscript. LI and AP coordinated the study and validated the results. All authors revised the manuscript and read and approved the final manuscript.

## Funding

Funding was ensured by the Ministry of Health.

## Conflict of interest

The authors declare that the research was conducted in the absence of any commercial or financial relationships that could be construed as a potential conflict of interest.

## Publisher's note

All claims expressed in this article are solely those of the authors and do not necessarily represent those of their affiliated organizations, or those of the publisher, the editors and the reviewers. Any product that may be evaluated in this article, or claim that may be made by its manufacturer, is not guaranteed or endorsed by the publisher.

## Footnotes

^1^ https://www.cnscbt.ro/index.php/situatia-la-nivel-global-actualizata-zilnic?limit=10&limitstart=0

^2^ https://vaccinare-covid.gov.ro/vaccinarea-SARS-CoV-2/procesul-de-vaccinare-in-romania/

^3^ https://www.who.int/publications/m/item/ECBS-Executive-Summary.IF.IK.TW-15_Dec_2020

^4^ https://www.corelaboratory.abbott/int/en/offerings/segments/infectious-disease/SARS-CoV-2-
